# Effects of Jianpi Qingchang decoction on the quality of life of patients with ulcerative colitis: A randomized controlled trial

**DOI:** 10.1097/MD.0000000000006651

**Published:** 2017-04-21

**Authors:** Yan-Cheng Dai, Lie Zheng, Ya-Li Zhang, Xuan Chen, De-Liang Chen, Zhi-Peng Tang

**Affiliations:** Institute of Digestive Disease, China-Canada Center of Research for Digestive Diseases, Longhua Hospital, Shanghai University of Traditional Chinese Medicine, Shanghai, China.

**Keywords:** Jianpi Qingchang decoction, quality of life, ulcerative colitis

## Abstract

This study aims to determine the effects of the Jianpi Qingchang decoction (JQD) on the quality of life (QOL) of patients with spleen deficiency and dampness-heat syndrome ulcerative colitis (UC).

A total of 120 active UC patients with spleen deficiency and dampness-heat syndrome were enrolled into this study. These patients were randomly divided into 2 groups: test group and control group (n = 60, each group). Patients in the test group were treated with JQD, while patients in control group were treated with 5-amino salicylic acid. After treatment for 8 weeks, differences in inflammatory bowel disease questionnaire (IBDQ) scores, short form-36 health survey questionnaire (SF-36) scores, and Sutherland Disease Activity Index (DAI) values were compared between these 2 groups to assess the QOL of patients.

Sutherland DAI scores decreased in both groups after the treatment, but the difference was not statistically significant (*P* < .05). However, the difference in bowel symptoms, systemic symptoms, total scores of the 4 IBDQ dimensions (physical function, bodily pain, vitality, and mental health), and total scores of the SF-36 questionnaires between these 2 groups were statistically significant (*P* < .05).

JQD can be used as supplementary and alternative therapy to relieve clinical symptoms in patients with mild to moderate active UC, and consequently improve their QOL.

## 1 Introduction

Ulcerative colitis (UC) is a chronic intestinal disease. However, its pathogenesis remains unclear. Its main clinical manifestations include diarrhea, mucopurulent bloody stool, tenesmus, and abdominal pain. UC can seriously impact the quality of life (QOL) of patients due to long disease duration, and its wide range of pathological changes and recurrence.^[1]^ At present, improving the QOL of patients, inducing and maintaining clinical remission and mucosal healing, and preventing complications are regarded as targets for UC treatment.^[2]^ Both symptom scores and mucosal healing have been addressed through clinical trials, allowing researchers and clinicians to gain a greater appreciation of the fact that many symptoms may not be driven by active inflammation. Hence, focusing only on immunomodulatory therapies would not fully serve the needs of patients. Furthermore, there is an emerging recognition on the importance of stress and psychological health in influencing QOL in symptom experience and treatment needs.^[3]^ Jianpi Qingchang decoction (JQD) is a treatment based on syndrome differentiation in patients with spleen deficiency and dampness-heat syndrome UC. Previous studies have found that JQD could be used to simply treat initial or mild UC, and reduce the Sutherland disease activity index (DAI) scores.^[4]^ Furthermore, JQD can upregulate the expression of glucocorticoid receptor-α in peripheral blood mononuclear cells, which improves hormone-dependent status in treating steroid-dependent UC.^[5]^ In addition, JQD can also suppress the activation of the nuclear factor-kappa B signaling pathway, and reduce the expression of cytokines in dextran sulfate sodium (DSS)-induced colitis in mice.^[6]^ Although JQD has a certain efficacy on patients with UC, commonly used objective indicators such as colonoscopy, the DAI and mucosal healing are not sensitive enough to reflect the patient's psychology, social ability, and other factors; and it cannot embody the feature that JQD treatment for UC is based on syndrome differentiation in Traditional Chinese Medicine (TCM).^[7,8]^ Previous studies have shown that complementary and alternative medicine can improve the QOL of patients with other diseases.^[9,10]^ Therefore, the aim of the present study was to investigate the effect of JQD on the QOL of UC patients, in order to objectively evaluate the clinical efficacy and safety of JQD for UC treatment.

## 2 Materials and methods

### 2.1 Subjects

This study is a single-center, randomized and controlled trial conducted at Long Hua Hospital, Shanghai University of Traditional Chinese Medicine, and is registered at the Chinese Clinical Trial Registry (ChiCTR-TRC-14004153). All participants provided a signed informed consent before the trial. Furthermore, the Ethics Committee of Long Hua Hospital approved the protocol for this experiment, and there were no significant changes during the experiment.

### 2.2 Inclusion criteria

Patients who had mild to moderate active UC based on the diagnostic criteria (classified using the Sutherland DAI value as reference).^[11,12]^ The TCM syndrome was spleen deficiency and dampness-heat syndrome, and these symptoms included abdominal distension and pain, relatively high frequency of stool, thin fecal matter, red and white sticky jelly-like stool or indigested food in stool, pale complexion, mental fatigue, pale tongue with greasy moss, and weak pulse.^[13,14]^ All patients were above 18 years old, and there were no limitations on gender. Patients did not have any concurrent disease in other systems that might seriously impact their QOL. All patients volunteered to sign an informed consent, had normal communication skills, and could complete the questionnaire alone or with the help of another person.

### 2.3 Exclusion criteria

Patients who refused to provide a signed informed consent for participating into the study; patients who had concurrent diseases in other systems that could seriously affect their QOL; patients who could not understand the questionnaire even with the explanation and help of others; patients who were under 18 years old and were allergic to multiple drugs; patients participating in other clinical trials at the time of the study.

### 2.4 Randomization and intervention

MS Excel 2007 was used to generate the random number table. Sequential numbers from 1 to 120 with 1 increment were selected and used to obtain random numbers for 120 times. Patients assigned to the first 60 numbers were included in the test group, and the remaining patients were included in the control group. Patients were assigned random numbers based on the treatment order, and the corresponding medicine was given based on the number. Patients in the test group were treated with JQD. (The 9 medicinal herbs used in JQD are shown in Table 1.) The patients in the test group were given 400 mL of the decoction, 200 mL bid po; while patients in the control group were given 1.0 g of 5-amino salicylic acid (5-ASA) qid po (commercial name: Huidi; Sunflower Pharmaceutical Group, Jiamusi Lu Ling Pharmaceutical Co, Ltd, Liaoning, China). The treatment duration for both the groups was 8 weeks.

**Table 1:**
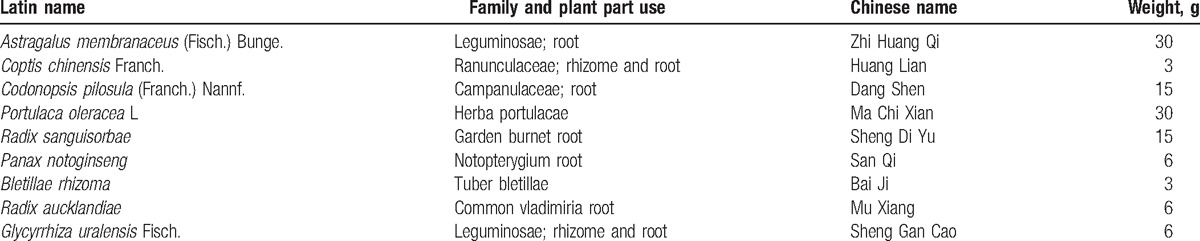
Composition of Jianpi Qingchang decoction.

### 2.5 Exit criteria

Patients whose symptoms worsened and whose Sutherland DAI scores increased by ≥30% during the treatment, compared with the baseline, were excluded from the study. Patients with serious adverse reactions during treatment, or patients who voluntarily quit or were found to be ineligible for the study by the investigators were also excluded from the study. If the patient's total medication was not within 80% to 120% of the total required amount of medication after the trial, the patient was removed from the study. Patients were excluded from the study due to the following reasons: personal request due to health considerations, or requested by the investigator.

### 2.6 Observation indices

General information: gender, age, and disease course. Primary efficacy evaluation indices: QOL scores before and after treatment, as evaluated by the inflammatory bowel disease questionnaire (IBDQ) and the short form-36 health survey questionnaire (SF-36).^[15]^ Secondary efficacy evaluation indices: DAI before and after treatment, as evaluated by Sutherland DAI values. A total score of ≤2 indicates symptom relief, a total score of 3 to 5 indicates mild activity, a total score of 6 to 10 indicates moderate activity, and a total score of 11 to 12 indicates severe activity.

### 2.7 Safety evaluation indices

Before and after the 8-week treatment mark, all patients underwent routine examinations, including blood and urine tests, stool tests, electrocardiogram examinations, liver function tests (alanine aminotransferase and aspartate transaminase), and kidney function tests (urea nitrogen and creatinine). Furthermore, the type, time of occurrence, frequency, duration, and severity of adverse reactions during the whole trial process were recorded and analyzed.

### 2.8 Statistical analysis

SPSS 18.0 was used for statistical analysis, and the quantified resource data were represented as means ± standard deviation (
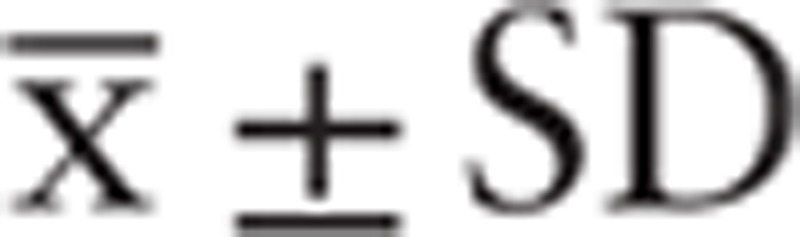
). The normality and homogeneity of variance were tested for all data. For data that revealed normal distribution and had homogeneity of variance, analysis of variance was carried out for comparisons between multiple groups. Least significant differences *t* was applied for internal group comparisons. Statistical significance was considered when the *P* value was <.05.

## 3 Results

### 3.1 Research population

**Figure 1:**
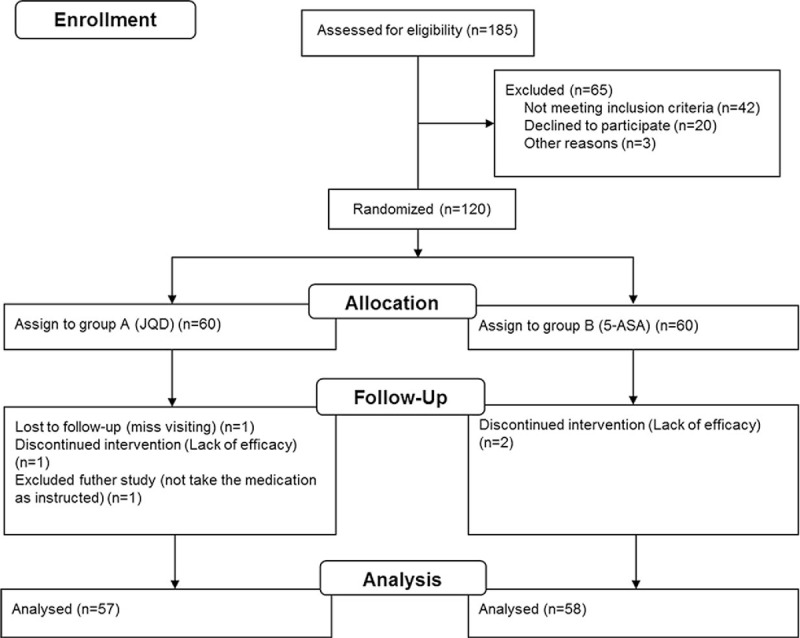
A total of 120 patients were recruited into the study and divided into 2 groups: test group and control group. Patients in the test group were treated with JQD, while patients in control group were treated with 5-ASA. In the test group, 1 patient left the study due to poor efficacy, 1 patient dropped out from the study for not following the instructed medication, and 1 patient was lost to follow-up. In the control group, 2 patients dropped out of the study due to poor efficacy. 5-ASA = 5-amino salicylic acid, JQD = Jianpi Qingchang decoction.

A total of 120 patients at the Outpatient Clinic or Ward of the Gastroenterology Department of Long Hua Hospital from January 2014 to June 2015 were recruited into the study. These patients were divided into 2 groups: test group and control group. In the test group, 1 patient left the study due to poor efficacy, 1 patient dropped out from the study for not following the instructed medication, and 1 patient was lost to follow-up. In the control group, 2 patients dropped out of the study due to poor efficacy (Fig. 1).

### 3.2 Baseline data

All patients completed the baseline screening. There was no statistically significant difference between these 2 groups in terms of baseline characteristics (age and gender), disease course, Sutherland DAI scores, total SF-36 scores, total IBDQ scores, and TCM single symptom evaluation (*P* > .05) at baseline visits. This indicates that the baseline data for these 2 groups were balanced and comparable (Table 2).

**Table 2:**
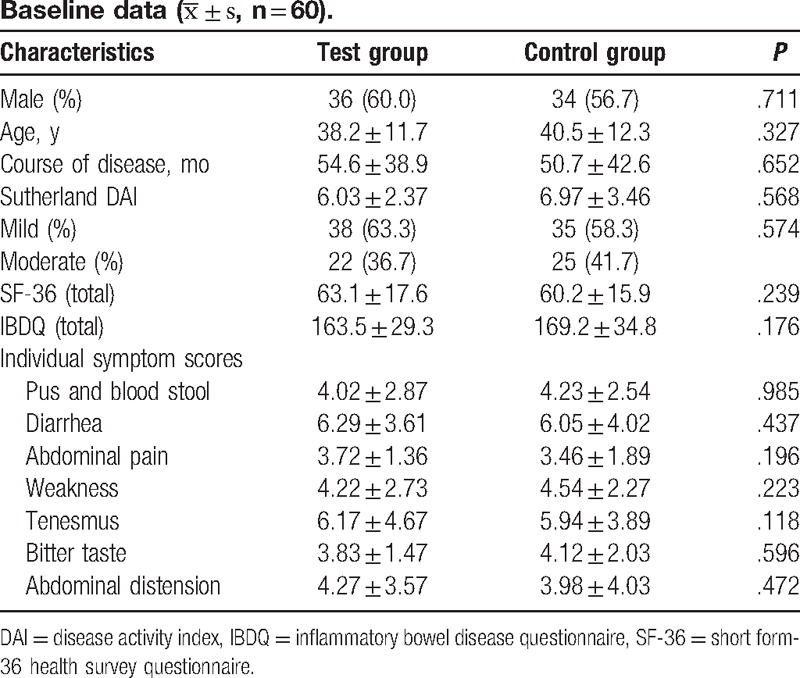


### 3.3 Analysis of primary outcomes

Total and dimensional IBDQ scores increased in both the test and control groups, compared to baseline (*P* < .05); and differences in systemic symptoms and total scores were statistically significant between these 2 groups (*P* < .05, Table 3). Total and dimensional SF-36 scores also increased in both the test and control groups before and after treatment (*P* < .05). When these 2 groups were compared, differences in the 4 dimensional scores, including physical function (PF), bodily pain (BP), vitality (VT) and mental health (MH), and the total scores were statistically significant (*P* < .05, Table 4).

**Table 3:**
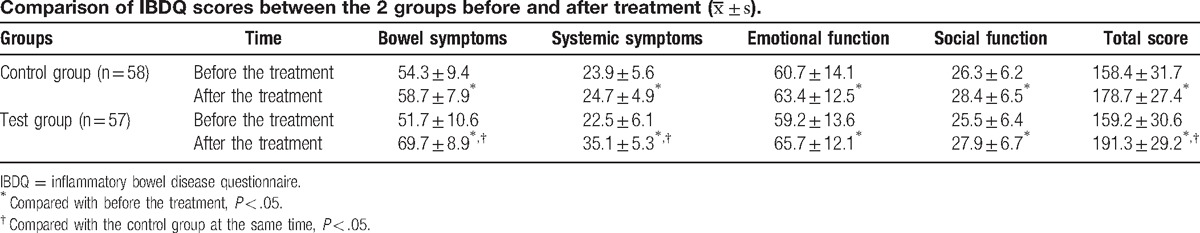


**Table 4:**
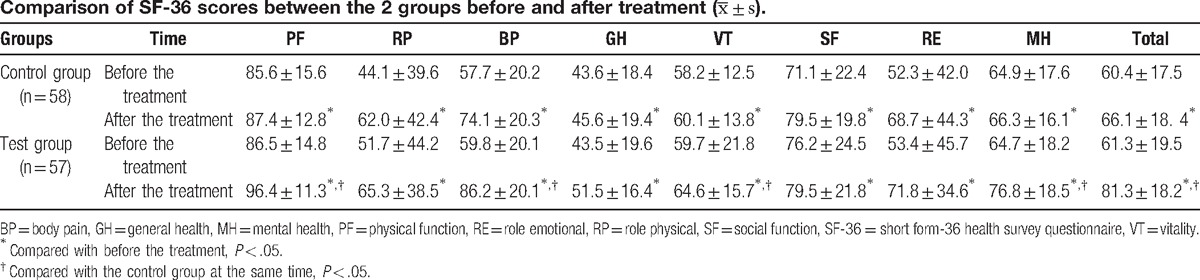


### 3.4 Analysis of secondary outcome

Sutherland DAI scores significantly decreased in both the test and control groups after treatment (*P* < .05), but the differences between these 2 groups were not statistically significant (*P* > .05, Table 5).

**Table 5:**
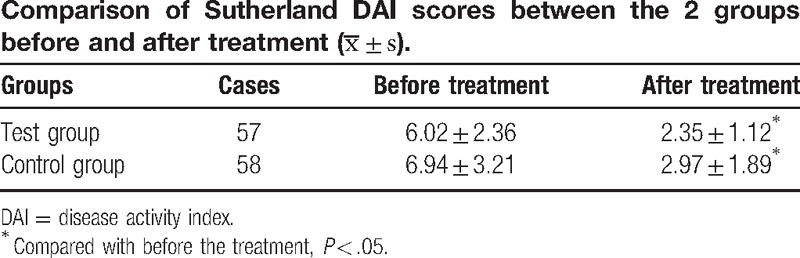


### 3.5 Safety analysis

The incidence of adverse events was similar in the test and control groups, which were 12 (20%) and 13 (21.7%), respectively. Most adverse events were mild to moderate such as nausea, fatigue, abdominal pain, dizziness, consciously fever, dyspepsia, and rash. Adverse events related to drugs are listed in Table 6. Patients in neither of the 2 groups had severe adverse events, and the liver and kidney functions of patients in these 2 groups did not exhibit any significant changes (*P* > .05).

**Table 6:**
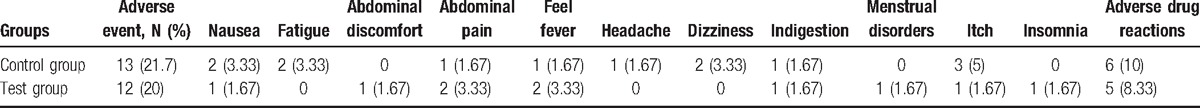
Adverse events and adverse drug reactions (cases [%], n = 60).

## 4 Discussion

Most patients with UC have continued negative emotions, such as anxiety and depression. Since UC is recurrent and refractory, its treatment is expensive and medications may have side effects, which may result in physical and psychological discomfort, consequently affecting the QOL of patients.^[16,17]^ At present, some scholars have considered that adverse mental and psychological factors may induce changes in brain-gut axis function, excite the autonomic nervous system, promote the release of various neurotransmitters, increase the activity of immune cells, and alter the interaction of bacteria and mucous membranes.^[18,19]^ Thus, these adverse factors result in the occurrence and development of UC, and exacerbate the feelings of discomfort of patients.^[20]^ In short, psychological and social factors, as well as medical expenses, can influence the QOL of UC patients, as well as their intestinal symptoms.^[21]^

5-ASA is the first line of treatment for mild to moderate UC. Previous studies have reported that the oral or enema administration of 5-ASA could relieve the clinical symptoms of UC and improve the QOL of patients with mild to moderate UC.^[22]^ The clinical symptoms of UC are similar to those of the dysentery in TCM. The pathogenesis of UC is spleen deficiency, which results in dampness, heat, stasis, and other pathogenic factors.^[8,13]^ Deficiency of the spleen may affect the digestive function of the large intestine, leading to poor appetite, abdominal distension, weakness, and increased frequency of bowel movements. Damp-heat may impair the collaterals of the large intestine, leading to stools containing pus and blood. Thus, UC treatment should regulate the qi that flows for strengthening the spleen and eliminating the dampness-heat syndrome. JQD was invented based on this principle: *Codonopsis pilosula* and radix Astragali could nourish the qi, Portulacaceae and *Radix sanguisorbae* could clear heat and dampness, and *Panax notoginseng* and Tuber *Bletilla striata* could promote blood circulation by removing blood stasis.^[23,24]^ Modern studies have found that *Astragalus membranaceus* (Fisch.) Bunge containing Astragalus polysaccharide had a beneficial immune regulatory effect on experimental colitis, which promoted the expression of T helper cell 1 (Th1) and T helper cell 2 (Th2)-specific transcription factors, but ultimately led to a shift toward the Th2 phenotype.^[25]^ Berberine, the main component of *Coptis chinensis* Franch, reduced the severity of chronic relapsing DSS-induced colitis by suppressing Th17 responses.^[26]^

This research had a single-center, randomized and controlled study design. Based on previous studies, the effects of JQD in patients with spleen deficiency and dampness-heat syndrome UC, as well as in their QOL, were observed by applying the IBDQ and SF-36 QOL scales, combined with Sutherland DAI scores. These results indicate that Sutherland DAI scores decreased in both groups after treatment, but the difference was not statistically significant. However, the 2 groups were significantly different with respect to bowel symptoms, systemic symptoms, the total score of the 4 IBDQ dimensions (PF, BP, VT, and MH), and the total score of SF-36.

JQD can improve the QOL of active UC patients with spleen deficiency and dampness-heat syndrome, which reflects the advantages of the individualized and differential treatment with JQD. In this case, change from the emphasis on subjective symptoms and the evaluation of traditional syndromes to objective data on the QOL scale can provide relatively objective evidence for the standardization and clinical evaluation of this syndrome in Chinese patients. Moreover, it can reflect the general health status, emotional roles, and other present situations of these patients, and provide a basis for the clinical treatment and efficacy evaluation to supplement the existing evaluation system without violating the theories in TCM. However, it should be further verified whether JQD is effective for UC patients with other TCM syndromes. This study had a small research population, and was a single-center clinical study. Experiments with large sample populations through multicenter clinical researches with follow-up observation should be conducted to validate the findings of this study, and to determine the physiological and pathological mechanism of JQD, with the aim of improving the QOL of UC patients.
